# Crystal and solution structural studies of mouse phospholipid hydroperoxide glutathione peroxidase 4

**DOI:** 10.1107/S2053230X16013686

**Published:** 2016-09-22

**Authors:** Robert Janowski, Sandra Scanu, Dierk Niessing, Tobias Madl

**Affiliations:** a Group Intracellular Transport and RNA Biology, Institute of Structural Biology, Helmholtz Zentrum München – German Research Center for Environmental Health, Ingolstädter Landstrasse 1, 85764 Neuherberg, Germany; b Institute of Structural Biology, Helmholtz Zentrum München – German Research Center for Environmental Health, Ingolstädter Landstrasse 1, 85764 Neuherberg, Germany; c Department of Chemistry, Technische Universität München, Lichtenbergstrasse 4, 85748 Garching, Germany; d Department of Cell Biology at the Biomedical Center, Ludwig-Maximilians-Universität München, Grosshaderner Strasse 9, 82152 Munich, Germany; e Institute of Molecular Biology and Biochemistry, Center of Molecular Medicine, Medical University of Graz, Harrachgasse 21/III, 8010 Graz, Austria

**Keywords:** phospholipid hydroperoxide glutathione peroxidase 4, reactive oxidative species, NMR spectroscopy, small-angle X-ray scattering

## Abstract

The mammalian glutathione peroxidase (GPx) family is a key component of the cellular antioxidative defence system. Within this family, GPx4 has unique features as it accepts a large class of hydroperoxy lipid substrates and has a plethora of biological functions, including sperm maturation, regulation of apoptosis and cerebral embryogenesis. In this paper, the structure of the cytoplasmic isoform of mouse phospholipid hydroperoxide glutathione peroxidase (O70325-2 GPx4) with selenocysteine 46 mutated to cysteine is reported solved at 1.8 Å resolution using X-ray crystallography. Furthermore, solution data of an isotope-labelled GPx protein are presented.

## Abstract 2

The crystal structure of mouse phospholipid hydroperoxide glutathione peroxidase 4 solved at 1.8 Å resolution and the first solution structural studies of a glutathione peroxidase protein are reported.

## 1. Introduction   

The mammalian glutathione peroxidase (GPx) family is a key component of the cellular antioxidative defence system (Burk & Hill, 1993 ▸). Its eight members (GPx1–GPx8) catalyse the reduction of reactive oxidative species (ROS) such as hydrogen peroxide (H_2_O_2_) or other alkyl hydroperoxides at the expense of reduced glutathione and/or other reductants (Burk & Hill, 1993 ▸; Tosatto *et al.*, 2008 ▸; Toppo *et al.*, 2008 ▸; Brigelius-Flohé & Maiorino, 2013 ▸). This enzymatic reaction involves either a cysteine or a selenocysteine as part of the catalytic tetrad.

GPx4 was first identified in pig liver (Ursini *et al.*, 1982 ▸) and has unique features among other members of the GPx family: it accepts a large class of hydroperoxy lipid substrates (Thomas *et al.*, 1990 ▸), shows a strong tendency for polymerization (Brigelius-Flohé, 1999 ▸; Imai & Nakagawa, 2003 ▸; Ursini *et al.*, 1999 ▸) and has several biological functions (Imai & Nakagawa, 2003 ▸; Sattler *et al.*, 1994 ▸), including sperm maturation (Ursini *et al.*, 1999 ▸; Ingold *et al.*, 2015 ▸), regulation of apoptosis (Nomura *et al.*, 1999 ▸) and cerebral embryogenesis (Borchert *et al.*, 2006 ▸).

The protein is expressed at low levels in most mammalian tissues, with larger amounts present in the testis (Brigelius-Flohé, 1999 ▸). Tissue-specific depletion of GPx4 results in several phenotypes in mice, including severe neurodegeneration and seizures (Seiler *et al.*, 2008 ▸; Wirth *et al.*, 2010 ▸), multi-organ thrombus formation (Wortmann *et al.*, 2013 ▸), as well as epidermal hyperplasia, dermal inflammatory infiltrate, dysmorphic hair follicles and alopecia (Sengupta *et al.*, 2013 ▸). Low testicular levels of GPx4 or expression of GPx4 single-point mutants are related to male infertility (Ingold *et al.*, 2015 ▸; Imai *et al.*, 2001 ▸). Very recently, GPx4 has been identified as a key regulator in a novel type of non-apoptotic cell death termed ferroptosis (Yang *et al.*, 2014 ▸; Friedmann Angeli *et al.*, 2014 ▸). However, most of the underlying molecular mechanisms remain enigmatic.

Among the three different isoforms present in nature, the 170-residue, 19.5 kDa monomeric cytosolic isoform is essential (Ho *et al.*, 1997 ▸). Its key feature is the catalytically active tetrad containing selenocysteine (Sec46), glutamine (Gln81), tryptophan (Trp136) and asparagine (Asn137) (Scheerer *et al.*, 2007 ▸; Tosatto *et al.*, 2008 ▸). Therein, Sec46 is activated and stabilized by Gln81 and Trp136 through a network of hydrogen bonds (Aumann *et al.*, 1997 ▸; Maiorino *et al.*, 1995 ▸), whereas the main role of Asn137 appears to be primarily in the initial oxidation step (Tosatto *et al.*, 2008 ▸).

With the crystallization of the human GPx4 selenocysteine-to-cysteine single-point mutant, the first insights into the unique properties of GPx4 were obtained (Scheerer *et al.*, 2007 ▸; PDB entries 2gs3 and 2obi). Nevertheless, given that most *in vivo* studies on the role of GPx4 in ROS regulation and its interaction network are currently carried out in mouse models, it is of advantage to obtain structural information for the mouse orthologue as well. Furthermore, it would be beneficial to complement the X-ray crystallographic studies of GPx4 with solution studies of the protein, for example by NMR spectroscopy and/or small-angle scattering, in order to set the stage for structural studies with ligands that might be difficult to co-crystallize and to obtain information on protein dynamics that possibly play an important role in GPx4 enzymatic activity (Goodey & Benkovic, 2008 ▸; Schnell *et al.*, 2004 ▸; Loria *et al.*, 2008 ▸). In this respect, it is noteworthy that no comprehensive solution NMR spectroscopic studies of glutathione peroxidases have been reported to date.

Here, we present a study of the expression, purification and determination of the crystal structure of the selenocysteine 46 to cysteine (U46C) mutant of mouse GPx4. We compare this structure with the previously published structures of human GPx4 (Scheerer *et al.*, 2007 ▸; PDB entries 2gs3 and 2obi) and present solution NMR spectra of isotope-labelled protein for the first time. Furthermore, we present small-angle X-ray scattering (SAXS) data indicating that GPx4 is monomeric in solution under reducing conditions. Our data comprehensively describe the three-dimensional structure of the mouse enzyme to 1.8 Å resolution and demonstrate that more extensive solution studies of the enzyme focusing on protein dynamics and GPx4–ligand interactions will be possible in the future. Furthermore, our solution data indicate that the strong tendency of GPx4 for polymerization is likely to be mediated by intermolecular disulfide-bond formation.

## 2. Materials and methods   

### 2.1. Macromolecule production   

The construct corresponding to the Met1–Leu170 (GPx4) region of mouse cytosolic phospholipid hydroperoxide glutathione peroxidase (O70325-2) with selenocysteine 46 mutated to cysteine was purchased from ATG:biosynthetics GmbH in a pUC cloning vector. The DNA sequence was codon-optimized for protein production in bacterial cells and flanked by NcoI and BamHI restriction sites. The coding region was cloned into a modified pETM-11 bacterial expression vector (kindly provided by Arie Geerlof, Protein Expression and Purification Facility, Helmholtz Zentrum München, Germany) which was derived from a pET-24d(+) vector (Novagen) by insertion of a *Tobacco etch virus* (TEV) protease cleavage site following an N-terminal hexahistidine and protein A tag. The GPx4 gene was amplified by PCR using T4 primers (New England Biolabs). The resulting PCR products and pETM-11 were double-digested with NcoI and BamHI enzymes (New England Biolabs) before ligation. The construct was verified by sequencing. For cloning purposes, a glycine residue was introduced between Met1 and Cys2. The amino-acid residue numbering of GPx4 corresponds to the full-length protein.

Uniformly (^13^C,^15^N) double-labelled GPx4 was produced in freshly transformed *Escherichia coli* DE3 cells. A single colony was inoculated in 20 ml Luria–Bertani medium with 25 mg l^−1^ kanamycin and cultured at 37°C until the OD_600_ reached 1.0. From this, an aliquot (1 ml) was added to (^13^C,^15^N-labelled) M9 minimal medium (100 ml) in which ^15^N-NH_4_Cl (2 g l^−1^) and ^13^C-glucose (3 g l^−1^) were the only sources of nitrogen and carbon for NMR isotope-labelling purposes, respectively (Cambridge Isotope Laboratories). The culture was incubated overnight at 37°C and shaken at 200 rev min^−1^. Fresh (^13^C,^15^N) M9 minimal medium was added to 1 l and the culture was grown under the same conditions until the OD_600_ reached 1.0. Protein expression was induced with 1 m*M* isopropyl β-d-1-thiogalactopyranoside at 20°C. The cells were pelleted after 4 h by centrifugation using a Fiberlite F9-6×1000 rotor in a Sorvall LYNX 6000 Superspeed centrifuge at 2000*g* for 20 min, resuspended in 40 ml lysis buffer consisting of 50 m*M* NaH_2_PO_4_, 300 m*M* NaCl, 10 m*M* imidazole, 1 m*M* tris(2-carboxyethyl)phosphine (TCEP) pH 8.0, 2.5 µg ml^−1^ DNase, 1 mg ml^−1^ lysozyme and protease-inhibitor mix (Serva). Note that the addition of TCEP was essential to avoid protein precipitation. Gpx4 is unstable in the reducing agents dithiothreitol and β-mercaptoethanol. Cell lysis was performed by sonication for 10 min at 70% power with 0.5 Hz cycles (Sonopuls, Bandelin). The cell lysate was separated by ultracentrifugation using a Thermo Scientific SS-34 rotor in a Sigma 6K15 centrifuge at 20 000*g* for 45 min at 4°C and histidine-tagged GPx4 was affinity-purified *via* Ni–NTA resin (Qiagen). TEV protease (5 µg ml^−1^) was added to the eluate and dialysed overnight at 4°C against 50 m*M* NaH_2_PO_4_, 300 m*M* NaCl, 10 m*M* imidazole, 1 m*M* TCEP pH 8.0. The GPx4 fragment was separated from the tag by a second affinity-purification step *via* Ni–NTA resin. GPx4 was loaded onto a size-exclusion column (Superdex 75 10/300 GL, GE Healthcare) equilibrated with 100 m*M* MES, 5 m*M* TCEP pH 6.5 using an ÄKTA pure FLPC system. The purity was estimated by SDS–PAGE to be 95%, with the yield of pure protein being 5 mg per litre of culture. Macromolecule-production information is summarized in Table 1 ▸.

### 2.2. Small-angle X-ray scattering   

All SAXS data were recorded on an in-house SAXS instrument (SAXSess mc^2^, Anton Paar) equipped with a Kratky camera, a sealed X-ray tube source and a two-dimensional Princeton Instruments PI·SCX:4300 CCD detector (Roper Scientific). The scattering patterns were measured with 180 min exposure times (1080 frames, each of 10 s) for several solute concentrations in the range from 1.75 to 5.0 mg ml^−1^. Radiation damage was excluded based on a comparison of individual frames of the 180 min exposures, where no changes were detected. A momentum-transfer range of 0.012 < *s* < 0.63 Å^−1^ was covered [*s* = 4π sin(θ)/λ, where 2θ is the scattering angle and λ = 1.542 Å is the X-ray wavelength].

All SAXS data were analysed with *ATSAS* v.2.5. The data were processed with *SAXSQuant* v.3.9 and de-smeared using *GNOM* (Svergun, 1992 ▸) and *GIFT* (Bergmann *et al.*, 2000 ▸). The forward scattering *I*(0), the radius of gyration *R*_g_, the maximum dimension *D*_max_ and the interatomic distance distribution function *P*(*r*) were computed with *GNOM* (Svergun, 1992 ▸) and *GIFT* (Bergmann *et al.*, 2000 ▸). The masses of the solutes were evaluated by comparison of the forward scattering intensity with that of a human serum albumin reference solution (molecular mass 69 kDa) and using Porod’s law.

To generate *ab initio* shape models, a total number of 50 models were calculated using *DAMMIF* (Franke & Svergun, 2009 ▸), aligned, and averaged using *DAMCLUST*. The *ab initio* shape models were aligned with the crystal structure of GPx4 determined here using *SUPCOMB* (Kozin & Svergun, 2001 ▸).

### 2.3. NMR spectroscopy   

The two-dimensional ^1^H,^15^N HSQC NMR spectrum of ^13^C,^15^N-labelled GPx4 was recorded on a Bruker AV III 900 spectrometer (Bruker, Rheinstetten, Germany) at 25°C. The NMR buffer used was 100 m*M* MES, 5 m*M* TCEP pH 6.5, and 5% D_2_O was added for the field frequency lock. The labelled protein concentration was 100 µ*M*. The NMR spectrum was acquired before the protein crystallized spontaneously in the NMR tube. The NMR spectrum was processed using Bruker *TopSpin* 3.2.

### 2.4. Crystallization   

The GPx4 protein crystallized spontaneously from a solution consisting of 100 m*M* MES, 5 m*M* TCEP pH 6.5 at a concentration of 2 mg ml^−1^ at room temperature. Rod-like, long, hexagonal crystals appeared in the 1.5 ml reaction tube. Crystallization information is summarized in Table 2 ▸.

### 2.5. Data collection and processing   

For the X-ray diffraction experiments, crystals were mounted in a nylon-fibre loop and flash-cooled to 100 K in liquid nitrogen. Diffraction data for GPx4 were collected on the X06SA beamline at SLS, Villigen, Switzerland using a PILATUS 6M detector at a wavelength of 0.99999 Å. The crystal diffracted to 1.8 Å resolution. The data set was indexed and integrated using *XDS* (Kabsch, 2010 ▸) and scaled using *SCALA* (Evans, 2006 ▸; Winn *et al.*, 2011 ▸). Intensities were converted to structure-factor amplitudes using *TRUNCATE* (French & Wilson, 1978 ▸). Table 3 ▸ summarizes the data-collection and processing statistics.

### 2.6. Structure solution and refinement   

The crystals of mouse phospholipid hydroperoxide glutathione peroxidase (GPx4) are isomorphous to the previously obtained crystals of the human homologue (PDB entry 2obi; Scheerer *et al.*, 2007 ▸). The proteins share 93% sequence identity. For the correct placement of the human homologue into the crystals of mouse GPx4, we performed molecular replacement with *MOLREP* (Vagin & Teplyakov, 2010 ▸) from the *CCP*4 suite (Winn *et al.*, 2011 ▸). Model building was performed in *Coot* (Emsley *et al.*, 2010 ▸). Refinement was performed in *REFMAC*5 (Murshudov *et al.*, 2011 ▸) using the maximum-likelihood target function including TLS parameters (Winn *et al.*, 2001 ▸). The final model is characterized by *R*_cryst_ and *R*_free_ factors of 0.151 and 0.193, respectively. Stereochemical analysis of the model was performed in *PROCHECK* (Laskowski *et al.*, 1993 ▸) and *MolProbity* (Chen *et al.*, 2010 ▸). It indicated that there are no residues with generously allowed or unfavourable backbone dihedral angles and that 99% of all residues are in the core region of the Ramachandran plot. The refinement parameters are shown in Table 4 ▸. All software was part of the *SBGrid* software bundle (Morin *et al.*, 2013 ▸). Atomic coordinates and structure factors have been deposited in the Protein Data Bank under accession code 5l71. Mouse and human GPx4 are isomorphous and show the same crystal packing.

## 3. Results and discussion   

**Figure 1:**
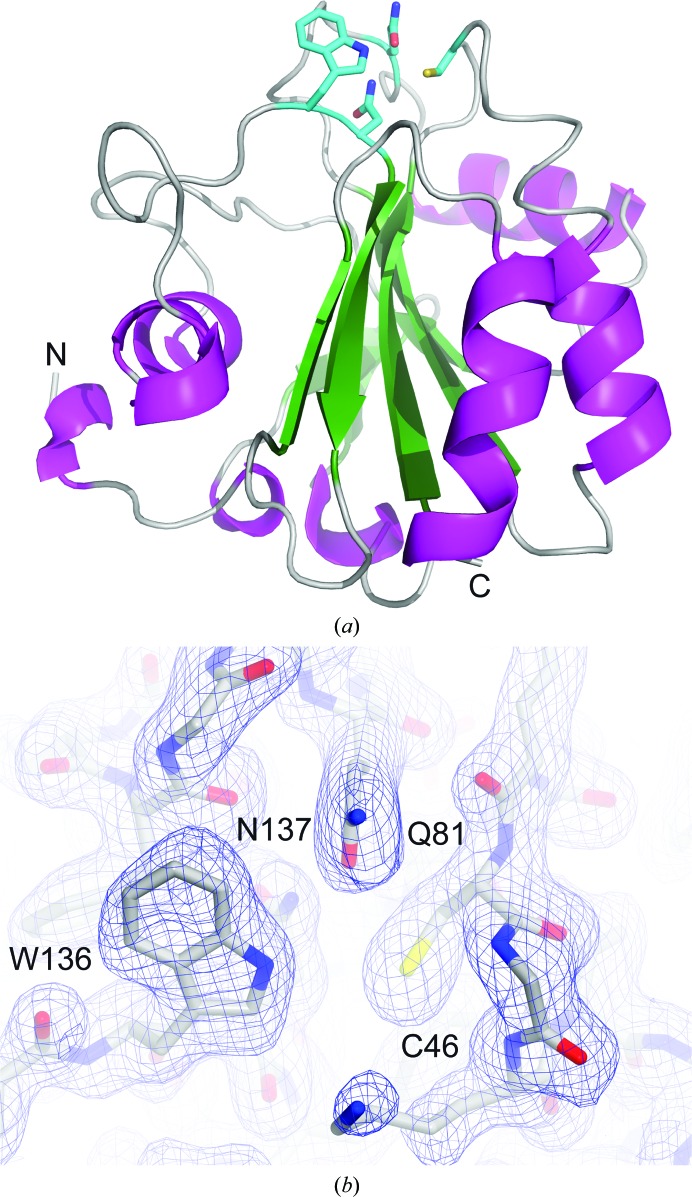
(*a*) The overall structure of mouse GPx4 shown as a ribbon. The secondary-structure elements are coloured as follows: helices, magenta; β-strands, green; loops, grey. The catalytic tetrad, located at the top of the structure, is shown in cyan. (*b*) shows a 2*F*_o_ − *F*_c_ electron-density map contoured at 1σ for the catalytic tetrad residues. (*a*) and (*b*) were prepared using *PyMOL* (v.1.8; Schrödinger) and *CueMol* (http://www.cuemol.org/en), respectively.

We successfully cloned, expressed and purified a fragment from Met1 to Leu170 of the cytoplasmic isoform of mouse GPx4 with selenocysteine 46 mutated to cysteine. Size-exclusion chromatography experiments indicated a monomeric state of the protein. GPx4 crystallized spontaneously in a 1.5 ml reaction tube at 2 mg ml^−1^ concentration at room temperature after acquiring the NMR spectrum. We took advantage of this fact and implemented X-ray crystallographic methods to solve the structure of this protein using single-crystal diffraction. To date there is no structure of mouse GPx4 protein available, and only two structures of its human homologue have been deposited in the Protein Data Bank [PDB entries 2obi (Scheerer *et al.*, 2007 ▸) and 2gs3 (Structural Genomics Consortium, unpublished work)]. Apart from this there are structures of GPx proteins from other organisms with lower sequence identity (for details see Supplementary Table S1; superposition of the selected structures is shown in Supplementary Fig. S1). For the structure solved in this study at 1.8 Å resolution (Fig. 1 ▸ *a*) the 2*F*_o_ − *F*_c_ electron-density map is clearly visible for residues Asp6–Leu170. The first six N-terminal residues, Met1, Gly (added for the purposes of cloning) as well as Cys2–Arg5, are not visible owing to disorder. Overall, the mouse GPx4 has the same fold as its human homologue. With a root-mean-square deviation (r.m.s.d.) of 0.21 Å for 165 superimposed C^α ^atoms, the structures can be considered to be isomorphous. They consist of four α-helices and several helical turns forming the surface of the protein, as well as seven β-strands organized in a mixed parallel and antiparallel manner forming the core of the structure (mouse GPx4 is shown in Fig. 1 ▸ *a*). The active site of the GPx4 enzyme consists of the catalytic tetrad Sec46, Gln81, Trp136 and Asn137. In this study selenocysteine 46 was mutated to cysteine. The active site is located at the top of the structure in the orientation presented in Fig. 1 ▸(*a*). A close-up of the tetrad is presented in Fig. 1 ▸(*b*) and Supplementary Fig. S2(*a*). The active-site cavity is surrounded by a number of positively charged amino-acid residues (Lys48, Arg80 and Lys135; Supplementary Fig. S2*b*). All of the cysteine residues are in the reduced form.

**Figure 2:**
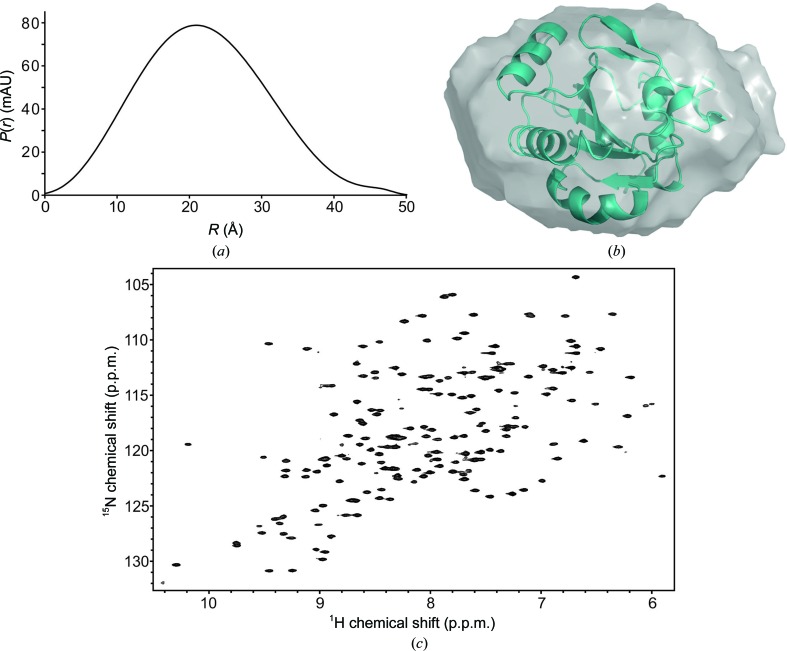
(*a*) The SAXS-based experimental radial density distribution shows that GPx4 adopts a compact conformation in solution. The following SAXS-based parameters have been derived: an apparent molecular mass of 20 kDa (calculated, 20 kDa), an *R*_g_ of 16.8 Å (16.5 Å calculated from the X-ray structure), *D*_max_ of 50 Å (50 Å in the crystal structure model), agreement of experimental scattering data with scattering data back-calculated from the crystal structure χ^2^ = 1.1. (*b*) Overlay of the SAXS *ab initio* model with the GPx4 crystal structure determined here. The model was calculated by *DAMMIF* using an average of 50 structures (*R*_f_ = 0.0032). The figure was prepared using *PyMOL*. (*c*) Two-dimensional ^1^H,^15^N HSQC NMR spectrum of ^13^C,^15^N-labelled GPx4. The excellent dispersion and quality of the NMR resonances are as expected for a well folded 20 kDa protein and indicate that GPx4 is monomeric in solution. The spectrum was recorded with 16 scans, an inter-scan delay of 1 s and 256 complex points in the indirect dimension.

Functioning as a structural protein, GPx4 appears to be involved in the formation of the mitochondrial capsule during sperm development. For this function, the formation of high-molecular-mass polymers has been reported to be important (Ursini *et al.*, 1999 ▸). Given that GPx4 contains several cysteine residues, this raises the question whether polymerization is mediated by intermolecular disulfide-bond formation or whether other intermolecular interactions mediate these interactions. To test whether GPx4 polymerization occurs in solution under reducing conditions, we carried out small-angle X-ray scattering (SAXS) and NMR spectroscopic analysis. Scattering curves recorded at several solute concentrations showed that GPx4 is monomeric in solution, as indicated by an apparent molecular mass of 20 kDa (calculated molecular mass of 20 kDa), an *R*_g_ of 16.8 Å (16.5 Å calculated from the X-ray structure) and a *D*_max_ of 50 Å (the corresponding dimension in the model of the crystal structure is 50 Å; Figs. 2 ▸ *a* and 2 ▸ *b*, Supplementary Fig. S3). Experimental scattering data and a SAXS-based *de novo* structural model are in excellent agreement with scattering data back-calculated from the crystal structure determined here (χ^2^ = 1.1). We acquired a two-dimensional heteronuclear single-quantum coherence (HSQC) NMR spectrum of uniformly ^13^C,^15^N-labelled Gpx4. This method provides a fingerprint of all amide bonds of a protein, which allows mapping of the protein structure and dynamics with single-residue resolution. In line with the SAXS data, two-dimensional ^1^H,^15^N HSQC NMR data recorded on ^13^C,^15^N isotope-labelled protein shows well resolved narrow NMR resonances, indicating that the protein is well folded and monomeric (Fig. 2 ▸ *c*). The formation of a GPx4 dimer or higher oligomers would be visible in form of extensive line broadening and disappearance of NMR signals and can be excluded.

In summary, with 93% sequence identity the structures of mouse and human GPx4 are isomorphous. Our solution data further show that mouse GPx4 is monomeric under reducing conditions. Furthermore, our solution NMR and SAXS studies demonstrate that more extensive solution studies of the enzyme focusing on protein dynamics and GPx4–ligand interactions will be feasible in the future.

## 4. Related literature   

The following references are cited in the Supporting Information for this article: Crow *et al.* (2004 ▸), Dimastrogiovanni *et al.* (2010 ▸), Gabrielson *et al.* (2012 ▸), Hall *et al.* (2009 ▸), Koh *et al.* (2007 ▸) and Krissinel & Henrick (2004 ▸).

## Supplementary Material

PDB reference: GPx4, 5l71

## Figures and Tables

**Table 1:** Mouse GPx4 production information

Source organism	*Mus musculus* (mouse), cytoplasmic isoform O70325-2
DNA source	ATG:biosynthetics GmbH
Forward primer	N/A
Reverse primer	N/A
Cloning vector	pUC57
Expression vector	pETM-11
Expression host	*E. coli* BL21 (DE3)
Complete amino-acid sequence of the construct produced†	M*G*CASRDDWRCARSMHEFSAKDIDGHMVCLDKYRGFVCIVTNVASQCGKTDVNYTQLVDLHARYAECGLRILAFPCNQFGRQEPGSNQEIKEFAAGYNVKFDMYSKICVNGDDAHPLWKWMKVQPKGRGMLGNAIKWNFTKFLIDKNGCVVKRYGPMEEPQVIEKDLPCYL

† In this construct, selenocysteine 46 (underlined) was mutated to cysteine. The glycine residue in italics is a cloning artefact.

**Table 2:** Crystallization of GPx4

Method	Spontaneous crystallization
Plate type	1.5 ml reaction tube
Temperature (K)	293
Protein concentration (mg ml^−1^)	2
Buffer composition of protein solution	100 m*M* MES, 5 m*M* TCEP pH 6.5
Composition of reservoir solution	None
Volume and ratio of drop	N/A
Volume of reservoir	N/A

**Table 3:** Data collection and processing Values in parentheses are for the outer shell.

Diffraction source	X06SA, SLS
Wavelength (Å)	0.99999
Temperature (K)	100
Detector	PILATUS 6M
Crystal-to-detector distance (mm)	320.02
Rotation range per image (°)	0.1
Total rotation range (°)	140
Exposure time per image (s)	0.1
Space group	*P*3_1_21
*a*, *b*, *c* (Å)	61.26, 61.26, 113.98
α, β, γ (°)	90, 90, 120
Mosaicity (°)	0.2
Resolution range (Å)	100–1.80 (1.85–1.80)
Total No. of reflections	175065
No. of unique reflections	23549
Completeness (%)	99.8 (99.9)
Multiplicity	7.4 (7.6)
〈*I*/σ(*I*)〉	21.6 (4.09)
*R*_merge_ (%)	6.0 (70.4)
CC_1/2_ (%)	97.7
Overall *B* factor from Wilson plot (Å^2^)	29.3

**Table 4:** Structure solution and refinement Values in parentheses are for the outer shell.

Resolution range (Å)	53.050–1.800 (1.847–1.800)
Completeness (%)	99.79 (99.88)
σ Cutoff	None
No. of reflections, working set	22298 (1600)
No. of reflections, test set	1251 (94)
Final *R*_cryst_	0.151 (0.216)
Final *R*_free_	0.193 (0.285)
No. of non-H atoms	
Protein	1348
Other	12
Water	185
R.m.s. deviations	
Bonds (Å)	0.03
Angles (°)	2.46
Average *B* factors (Å^2^)	
Protein	37.0
Ramachandran plot	
Most favoured (%)	99
Allowed (%)	1
